# Negative Campaigning and the Logic of Retaliation in Multiparty Competition

**DOI:** 10.1177/1940161215626566

**Published:** 2016-01-29

**Authors:** Martin Dolezal, Laurenz Ennser-Jedenastik, Wolfgang C. Müller

**Affiliations:** 1 University of Vienna, Vienna, Austria

**Keywords:** negative campaigning, political communication, multiparty competition, Austria

## Abstract

The extant literature has demonstrated that the *logic of retaliation* is a core feature of negative campaigning. Attacks by one side induce counterattacks by the other. Yet most research on the interactive nature of negative campaigning is limited to two-party competition and provides little theoretical justification for why political actors should respond to attacks with counterattacks. The present paper addresses these research gaps. We argue that the negativity bias in human information processing and the zero-sum nature of elections make retaliation a rational strategy. Importantly, these arguments also imply that retaliation may not be the only plausible response to attacks in multiparty systems. Rather, parties may prefer to react to attacks from one competitor by attacking another. To grasp empirically how being attacked and attacking are related, we conduct a highly disaggregated time series analysis of such instances while controlling for other factors that may influence actor behavior. Our analyses draw on several thousand party press releases issued during three national election campaigns in Austria, a typical European multiparty system. They show that retaliation is an important strategy also in multiparty politics. Yet in such context, parties do not exclusively follow a tit-for-tat approach but rather display more complex patterns of attack behavior.

## Introduction

Extant research has identified retaliation as one important driver of negative campaigning (e.g., Damore 2002; Druckman et al. 2010; Lau and Pomper 2004). Candidates who are attacked by their opponents often respond in kind—not least as this is what political practitioners canonically recommend (Theilmann and Wilhite 1998; Trent et al. 2011). Yet the study of the *logic of retaliation* remains incomplete in several respects: First, it lacks a clear theoretical argument as to why retaliation is preferable to ignoring attacks or responding with positive messages about oneself. Second, empirical analyses of retaliation often rely on aggregate data at the level of campaigns and thus find it difficult to demonstrate or at least approach causality. Third, it is not clear to what extent the insights from the best-researched elections, those in the United States, travel to systems with multiparty competition, proportional electoral systems, and coalition governments.

The contribution of this article is to address these three shortcomings. Theoretically, we build on the evidence of negativity bias in human information processing (Ito et al. 1998; Rozin and Royzman 2001). We argue that in combination with the logic of elections as zero-sum games, negativity bias makes retaliation a dominant strategy in two-party, winner-takes-all competition. Yet we also show that this argument may not necessarily generalize to other contexts. This discussion results in two hypotheses that capture specific aspects of the logic of retaliation, both in general terms and with respect to the context of multiparty competition. Empirically, we improve on existing research designs by conducting the most high-resolution time series analysis of attacks and counterattacks to date. As regards the political context, our analysis covers three national elections in Austria (2002, 2006, and 2008), a typical European multiparty system with proportional representation (PR) and coalition governments. Our study thus also contributes to the emerging field of research in negative campaigning in multiparty systems (Nai and Walter 2015).

Our main finding is that although multiparty competition allows for alternative strategies, the logic of retaliation is empirically strong. Once attacked, parties are likely to pay back the attacker in kind. Yet we also find evidence for more complex interactions, with the targets not turning against the sender but against parties not originally involved in the attack. This finding lends further support to the notion that multiparty competition and PR are crucial context factors that analyses of negative campaigning need to take into account.

## The Logic of Retaliation in Negative Campaigning

Scholars have amassed a huge wealth of research on negative campaigning. Explanations of its causes have largely focused on structural and performance-related factors. Incumbency, ideology, partisanship, campaign resources, performance in the polls, closeness of a race, and candidate gender, among others, have all been hypothesized to affect the tendency to “go negative” (Lau and Pomper 2004; Lau and Rovner 2009). Although numerous studies find these factors to have an impact on parties’ and candidates’ strategies, they tend to take a very aggregated and static view of campaigns. In a more disaggregated and dynamic perspective, campaigns might be conceived of as a stream of interaction where parties and candidates continuously respond to various stimuli and do so in strategically differentiated ways.

### Retaliation in the Extant Literature

How do parties react to attacks? Parties may simply neglect or repudiate them by defending the issue position or the candidate under attack without directly confronting the opponent. But they may also resort to a counterattack, as political consultants typically recommend (Trent et al. 2011). This seems to be intuitive: “If we had to choose one hypothesis [to explain negative campaigning] that we were most certain would be supported by the data, this would be our bet” (Lau and Pomper 2004: 33). The only exception to this rule might be attacks by weak candidates, which may safely be neglected (Theilmann and Wilhite 1998; Trent et al. 2011).

Although they are typically recommended as responses to attacks, counterattacks might also backfire. Experimental research indicates that both target and attacker incur substantial favorability losses. However, while respondents overtly condemn attacks, they also show higher levels of spontaneous conformity with a candidate who punches back (Carraro et al. 2012; see also Roddy and Garramone 1988). Furthermore, counterattacks can restore electoral support for the target to almost preattack levels (Craig et al. 2014). These results thus provide a rational foundation for counterattacks.

Empirical evidence based on observational research is rather thin. Only a small number of nonexperimental studies have examined whether the amount of attacks deployed by one party is dependent on how much it is attacked by its opponents. Some results are unambiguous and show a strong effect of previous attacks (Damore 2002; de Nooy and Kleinnijenhuis 2013; Druckman et al. 2010; Haynes and Rhine 1998; Lau and Pomper 2001, 2004). Other studies report mixed findings (Ridout and Holland 2010). Kahn and Kenney (1999), by contrast, did not find the expected association at all.

### Why Retaliate? Zero-Sum Elections and Negativity Bias

With respect to the theoretical foundation, the logic of retaliation appears to be so intuitive that its plausibility does not seem to require a great amount of justification. Researchers often refer to common wisdom, the advice of political consultants (Lau et al. 1999), or do not explain the rationale at all. If they do, two closely linked arguments are typically presented: First, a candidate who does not counterattack “create[s] the image that he [is] ineffectual and indecisive” (Ansolabehere and Iyengar 1995: 117). Second, while negative campaigning is indeed often disliked,^1^ mass media and voters are more likely to regard counterattacks as legitimate (Krupnikov and Bauer 2014).

To provide a more solid theoretical foundation, our argument builds on two premises. First, elections are zero-sum games. All gains and losses in vote shares sum to zero. It is therefore the *relative popularity* of parties that counts. Second, negative messages weigh heavier in human information processing than positive ones. If attacks are believed to have a negative net effect on the target, the loss in relative popularity can only be made up for by retaliating against the attacker.

The zero-sum logic obviously applies to two-party competition but is true even in multiparty systems. Here, one party’s loss is not automatically a win for any specific opponent, but it is certainly a gain for *some* competitor. What matters is thus not how voters evaluate parties in absolute terms but how parties stack up against each other. The vote-seeking imperative therefore dictates that parties try to maximize their support in the electorate *relative to that of others*.

With respect to our second premise, many (if not all) politicians and strategists believe that negative messages have an effect on voter evaluations, which is disadvantageous for the target even though the direct evidence that negative campaigning yields a net benefit for the attacker is rather thin (Lau and Pomper 2004; Lau et al. 1999; Lau et al. 2007). However, even if political operatives believe that attacks are harmful for the target, why should they choose to respond in kind instead of compensating the assumed losses with positive messaging about their own party? We argue that the prime obstacle to choosing a positive response over retaliation lies in the asymmetric impact that negative and positive messages have on evaluative processes—a phenomenon denoted as *negativity bias* (Rozin and Royzman 2001). As Ito et al. (1998) show, negativity bias is already present at very early stages of the evaluative process. Furthermore, negative messages are more influential than positive ones as they are more likely to be believed (Hilbig 2009). Thus, in many dimensions, “[b]ad is stronger than good” (Baumeister et al. 2001).

Negativity bias also applies to political messaging (Meffert et al. 2006).^2^ The most important implication in this regard is that negativity increases the news value of political messages (Groeling 2010; Lengauer et al. 2011). Given that the empirical material in our analysis comes from press releases, it is crucial to consider not only how voters will be influenced by party messages but also how the media may respond to campaign communication.

All else being equal, attacks and conflict frames are more newsworthy than noncontroversy (Harcup and O’Neill 2001; Semetko and Valkenburg 2000). In competing for the attention of journalists, parties therefore have incentives to respond to negative messages about themselves with negative messages about their competitors. Otherwise, their message may not attract as much media attention as the opponents’ initial attack.

The increasing importance of direct communication through social media notwithstanding, most campaign communication is still delivered through traditional media channels. Political actors thus do not only have to consider voter responses when drafting messages, but they also have to anticipate the process of journalistic news selection and adapt their behavior to the media logic.

This suggests that parties and candidates who are attacked may not find themselves able to reverse the negative effect of that attack by focusing on positive messages about themselves. As *bad* weighs heavier than *good* in the minds of voters and journalists, the damage resulting from an attack can only be compensated by responding in kind. Thus, targets of negative campaigning may be able to make up *relative* losses by retaliating and thus lowering the electorate’s perception of their opponent. The most straightforward way to offset the damage is to inflict a similar cost on the political opponent. Responding to an attack with a counterattack thus emerges as the dominant strategy. This is our first hypothesis:

**Hypothesis 1 (H1):** An attack from party A on party B raises the probability of a counterattack from party B on party A.

A counterattack is defined as an attack by the original attack’s target shortly after having been hit and directed against the attacker. This logic of retaliation is perfectly suited for two-party races and elections following a winner-takes-all logic. Multiparty races with PR, by contrast, might follow a different logic. Here, our understanding of negative campaigning remains limited as the existing studies focus mostly on the United States. Notwithstanding the importance of U.S. campaign style as international model generator, these studies also reflect the specifics of this country’s political system. This clearly applies to studies dealing with retaliation in two-party races such as elections to the U.S. presidency and congress (Damore 2002; Druckman et al. 2010; Kahn and Kenney 1999; Lau and Pomper 2001, 2004). However, even analyses covering multicandidate races such as nomination campaigns or primaries (Haynes and Rhine 1998; Ridout and Holland 2010) are not mirror pictures of PR systems due to the electoral system’s winner-takes-all logic.

### Moving to Multiparty Competition

Research on negative campaigning in (European) multiparty competition has so far not addressed the dynamics of campaign interactions. The only exception is the study of de Nooy and Kleinnijenhuis (2013) who find no evidence that Dutch politicians directly respond to attacks with counterattacks. Rather, political actors attack the allies of their attackers, but this result may in part be due to the fact that the study does not aggregate individuals into parties.

As argued above, the zero-sum logic also applies to multiparty competition, and the logic of retaliation (H1) might be dominant even in such races. Yet there are several important reasons why direct retaliation against the sender may not always be the most preferred option for the targeted party in multiparty systems.

First, there is often a stark asymmetry in size between sender and target. Larger parties have incentives to ignore smaller ones and instead focus on rivals within their “weight category,” simply because the electoral impact of attacking a smaller party is likely to be limited (Skaperdas and Grofman 1995; Walter 2014). Also, large mainstream parties may want to define the campaign’s center of gravity and not let it be captured by niche parties and niche issues (Meyer and Wagner 2013). Rather than merely absorbing the hits from smaller parties, larger parties may choose to react by attacking their main rivals to compensate the relative losses that they have incurred from being attacked.

Second, elections typically produce minority situations that necessitate the formation of a coalition government. Parties therefore need to be strategic about which of their competitors to attack, given that they may need partners after the election (Elmelund-Præstekær 2008; Walter and van der Brug 2013; Walter et al. 2014). A party’s goal then may not simply be to maximize its vote share but rather to maximize its bargaining power in postelection negotiations and therefore to maximize the number of potential viable coalition governments that it is part of. For example, if one right-wing party attacks another, retaliation may produce some intra-bloc voter exchange without expanding the overall prospects of a right-wing majority. The better strategy may be to react to attacks by attacking those rivals from which voters need to be drawn to produce the preferred coalition.

Third, a party may react to attacks by targeting whichever party it sees as its main competitor based on current poll ratings. Skaperdas and Grofman (1995), for instance, predict that parties in three-way competition never attack the weaker of their two opponents—this formal model assumes single-member districts, though. Still, even in multiparty PR systems, it may be rational for the runner-up to always attack the leading party (e.g., when finishing first confers an advantage in the government formation process). However, similar reasoning applies to smaller parties, as coming in just ahead of a competitor may make a significant difference in terms of postelection bargaining power.

A specific characteristic of multiparty competition is heightened competition for media attention. Given time and space constraints, it is more difficult to cover the policy statements and campaign messages of seven parties than of two. In instances where—for whatever reason—direct retaliation is not the preferred option, this makes reacting to attacks with positive issue or valence messages an unattractive option, as such messages are even less likely to prevail than they would be under two-party competition. The best reaction in such cases is then to attack a third party, as such a message will be of higher news value.

These arguments illustrate that attacks need not always trigger retaliation but can sometimes lead the targeted party to direct negative messages toward a third actor. Attacks by one party against another may simply lead the attacked party to reinforce its strategy of attacking whichever competitor they have singled out as their preferred target anyway. While one may object that it is difficult to ascertain in such instances whether it was the first attack that caused the second, we rely on extremely close temporal sequence and thousands of observations to examine whether the correlations in attack patterns conform to our assumptions.

To illustrate our theorizing more clearly, consider a party system with five parties A to E. Direct retaliation (H1) looks like this (arrows denote temporal sequence):

B attacks A→A attacks B

However, as it becomes clear from the examples above, other forms of interaction are also possible. These interactions involve more than two parties:

[C or D or E]attacks A→A attacks B

B attacks A→A attacks[C or D or E]

Here, party A chooses to attack a party other than the original instigator. Assume, for instance, that it may have been A’s strategy to attack B all along and that, for reasons such as those outlined above, the party would prefer not to be dragged into conflicts with other parties. So the way in which A reacts to an attack by C, D, or E is to reinforce its attacks on B. This type of interaction is captured by the two hypotheses below. H2a outlines the scenario in which party A always prefers to attack B and therefore reacts to attacks by other parties by targeting B. Under H2b, party A prefers to ignore attacks from B and reacts by attacking some other party.^3^

**Hypothesis 2a (H2a):** An attack on party A by *parties other than B* increases the probability of an attack of A on B.
**Hypothesis 2b (H2b):** An attack on party A by party B increases the probability of an attack of A on *parties other than B*.

## Empirical Strategy

### Case Selection

This study focuses on Austria, a typical West European parliamentary democracy with a PR electoral system, multiparty politics, coalition governments, and a democratic-corporatist media system (Hallin and Mancini 2004; Plasser and Lengauer 2010). While negative campaigning has been an important feature of elections throughout the postwar period, systematic studies are mostly missing (see Dolezal et al. 2015a).

In the present paper, we analyze three of the most recent general elections (2002, 2006, and 2008). In this period, the party system has included the two parties dominating postwar politics, the Social Democrats (Sozialdemokratische Partei Österreichs [SPÖ]) and the Christian democratic People’s Party (Österreichische Volkspartei [ÖVP]), the Greens (Die Grünen – Die Grüne Alternative), and the populist radical right Freedom Party (Freiheitliche Partei Österreichs [FPÖ]) plus its breakaway, the Alliance for the Future of Austria (Bündnis Zukunft Österreich [BZÖ]).

In terms of government formation, the by far most frequent coalition type in postwar Austria has been a grand coalition of the SPÖ and ÖVP. Yet in the elections studied, three different governments were in office, ÖVP-FPÖ (2002), ÖVP-BZÖ (2006), and SPÖ-ÖVP (2008), the first and the last of which were terminated in conflict and resulted in early elections. As a general pattern, Austrian parties act very cohesively and do not make public their coalition preferences before the election but sometimes commit *not* to form particular coalitions (e.g., the SPÖ has ruled out the FPÖ as a coalition partner since 1986).

### Data Source

Analyzing negative campaigning with a special focus on parties’ dynamic interaction requires data that capture not only which parties use these strategies and whom they attack. We also need the exact timing of party messages to infer patterns of attacks and counterattacks empirically—information not all sources provide. Our analysis is based on parties’ press releases. While common as a data source in agenda-setting studies (Brandenburg 2002; Tedesco 2005), press releases have hardly been used to analyze negative campaigning.^4^ This is a missed opportunity as they are a great source for observing parties’ campaign behavior and addressing our specific research question for two main reasons:

First, press releases are under the direct control of the sender. This is an obvious but important advantage compared with other modes of communication used by scholars so far. The analysis of media reports, for instance, does not capture party behavior directly but observes what journalists write about it (Elmelund-Praestekaer and Molgaard-Svensson 2014; Hansen and Pedersen 2008). Journalists may interpret actions by parties in a way that was actually not intended. Patterns of attack and retaliation, for example, might therefore not always be reported accurately.

Second, press releases are issued frequently and continuously during the campaign. Among all traditional means of political communication, this one allows for the quickest responses. The fact that every press release is traceable to an exact date and time also allows for precise sequencing. We can therefore analyze whether parties respond immediately to relevant stimuli such as attacks.

Contrary to media reports and televised debates, advertisements are also under the full control of parties and—in some contexts—occur at a high frequency. Especially TV spots that have become almost synonymous with political advertising, but also ads in newspapers and party posters have been widely used to study negative campaigning (e.g., Ansolabehere and Iyengar 1995; Elmelund-Præstekær 2008, 2010; Geer 2006; Hansen and Pedersen 2008; vanHeerde-Hudson 2011; Walter 2014; Walter and Vliegenthart 2010). In Austria, however, TV spots are not relevant as legal provisions prevent parties from buying airtime in the ORF, the still dominant public broadcast station (Plasser and Plasser 2002). By contrast, ads and posters are highly relevant, but they do not allow for a dynamic analysis as parties typically use the same design for several days or even weeks.

To be sure, there are some caveats about using press releases. The most relevant for the present purpose stems from the fact that they are a tool actors use to attract media attention. Parties may therefore adapt their message to journalistic selection criteria giving more weight to conflict and attacks. The overall level of negativity displayed by press releases should therefore not necessarily be considered representative of other campaign communication. However, we expect no bias in favor of our hypothesis from this fact, as heightened negativity should be present equally across parties.

Our study is based on all press releases sent by parties and candidates within the final six weeks of each campaign. We obtained them from the Austrian Press Agency’s (APA) Web site www.ots.at. We discard releases by low-ranking politicians (e.g., local representatives) as we are only interested in the national parties’ strategies. However, all candidates on party lists are regarded as relevant actors.

The remaining 6,750 press releases are coded using a relational method of content analysis that links actors to issues and/or other actors. A variable called *predicate* connects them and records whether their relation is positive (1), negative (−1), or neutral (0). This method goes back to the work of Kleinnijenhuis and his collaborators (e.g., Kleinnijenhuis and Pennings 2001) and was also used by Kriesi et al. (2006, 2008; Kriesi et al. 2012). The Austrian National Election Study (AUTNES) has further developed this approach and employs it for the analysis of various kinds of political text (see Dolezal et al. 2014; Dolezal et al. 2015b).

Following the standard approach in the literature (e.g., Geer 2006: 23), we define negative campaigning as any statement that is critically directed at a competitor. In our data, this refers to every actor–actor relation with a negative predicate whenever the actor addressed is a competing party or a representative of such a party. Table 1 shows the overall number of press releases and the share of attacks. As the BZÖ (an FPÖ split-off) only dates from 2005, there are fewer parties in 2002.

**Table 1.** Press Releases: Total Numbers Coded and Shares of Attack Releases.

Year	SPÖ	ÖVP	FPÖ	BZÖ	Greens	Total
Total number						
2002	1,063	672	123	—^a^	190	2,048
2006	1,049	477	261	297	167	2,251
2008	834	695	397	303	222	2,451
Attack releases						
2002	44.0%	33.7%	41.5%	—^a^	43.7%	40.7%
2006	41.6%	50.1%	55.9%	47.8%	55.1%	50.1%
2008	28.9%	45.3%	38.3%	45.9%	43.2%	40.3%

*Source.* AUTNES, only parties with representation in parliament included.

SPÖ = Sozialdemokratische Partei Österreichs, social democrats; ÖVP = Österreichische Volkspartei, Christian democrats/conservatives; FPÖ = Freiheitliche Partei Österreichs, populist radical right; BZÖ = Bündnis Zukunft Österreich, populist radical right; Greens = Die Grünen – Die Grüne Alternative.

a. The BZÖ was founded in 2005.

As mentioned above, counterattacks refer to the original actor–actor relation and are identified by sequence and extreme temporal closeness: Responses have to occur within the next one-hour interval after the original attack. Naturally, even an immediate response does not necessarily include a reference to the issue or candidate attribute the attacker referred to as we focus on the actors at the level of parties. Our identification strategy thus is in line with advice by political consultants who often argue that an attacked candidate should not address the issue mentioned by the opponent but rather seek to change the topic. Moreover, also using the level of parties is in line with parties’ actual behavior. A high-ranking politician, for instance, a cabinet minister, under attack from, let’s say, an MP is not likely to respond in person as this would enhance the status of the attacker.

### Data Structure

To capture the interactions posited in the hypotheses, we structure our data such that each observation represents one directed party dyad at a specific point in time during the campaign. Each directed party dyad consists of a sender and a target. In addition, we create a dichotomous indicator that records whether there was an attack by the sender on the target in each hour between 8 a.m. and 12 p.m. in the six weeks prior to the day of the election.^5^ This will be the dependent variable in the statistical models. We use the same information to create lagged indicators of attacks from the target on the sender as our central independent variables. Although it is still possible that interactions between parties occur at an even faster pace and thus escape our analysis (e.g., an attack sent out at 11.15 and a response at 11.40), using hours as the units of observation allows for many of the crucial interactions to be picked up.^6^

The data structure requires the use of binary time-series–cross-section (BTSCS) models. To capture time dependency in the data, we employ the technique proposed by Beck et al. (1998). In addition, we include four substantive control variables:

First, we include predictors for TV debates, which, in Austria, feature all possible pairs of party leaders. We assume that the televised confrontations raise the level of negativity between the two parties involved in the debate.

Second, we account for the ideological distance between sender and target (using data from the Chapel Hill expert surveys, see Bakker et al. 2015). In this regard, the literature generates expectations in two directions (Haynes and Rhine 1998; Ridout and Holland 2010; Walter 2014): Proximity makes parties likely partners—which should dampen negative campaigning—but at the same time, these parties should also compete for the same pool of voters—which should make negative campaigning more likely.

Third, we account for a core feature of multiparty systems: the need to form coalitions. Notwithstanding the vagueness in stated party coalition preferences in Austria, the number of potential coalitions is actually quite limited. Drawing on coalition theory, we assume ideological distance (de Swaan 1973; Martin and Stevenson 2001) and cabinet incumbency (Franklin and Mackie 1983; Golder et al. 2012) to be useful predictors of politicians’ expectations for the postelection period. In addition to ideological distance, we therefore also include a dummy variable for party dyads comprised of two government parties.

Fourth, we account for the size ratio between sender and target, as we expect larger parties to be targeted more frequently, while smaller ones will often be ignored. Finally, random effects pick up unobserved heterogeneity at the party-dyad level (fixed effects models are shown in the online appendix).

Descriptive statistics of all independent variables are shown in the online appendix. There, we also explain how we address the time structure in the data. The number of observations in our data is a function of the number of directed party dyads (twenty in 2008 and 2006, twelve in 2002) and the number of hours and days in the campaign (six weeks = forty-two days, 8 a.m. to 12 p.m. = sixteen hours). In addition, the use of lagged variables reduces the number of observations in the analysis.

## Analysis

Table 2 presents the BTSCS regressions modeling dyadic attack and response patterns in the three campaigns. The dependent variable is dichotomous and records whether there was an attack from the first party in the dyad (the sender) specifically directed at the other party in the dyad (the target).

**Table 2.** Explaining Attacks on Target within Dyad (Direct Retaliation).

	Hypothesis	2002	2006	2008
Attack from other party in dyad (t − 1)	H1	0.588***	0.387**	0.424***
		(0.134)	(0.126)	(0.123)
Attack from any party outside dyad (t − 1)	H2a	0.039	0.162	0.189^†^
		(0.137)	(0.117)	(0.111)
TV debate		0.696***	0.146	0.726***
		(0.172)	(0.139)	(0.119)
Left–right distance (sender–target)		0.104	−0.147	−0.045
		(0.195)	(0.106)	(0.067)
Coalition parties		−0.670	−0.666	2.044***
		(0.889)	(0.843)	(0.450)
Size ratio (sender–target)		0.097	−0.140^†^	−0.447***
		(0.254)	(0.074)	(0.110)
Hour		9.587***	7.386***	7.784***
		(0.831)	(0.638)	(0.607)
Hour (squared)		−0.634***	−0.497***	−0.518***
		(0.055)	(0.043)	(0.040)
Hour (cubed)		0.013***	0.010***	0.011***
		(0.001)	(0.001)	(0.001)
Day		0.005	0.008*	0.001
		(0.004)	(0.004)	(0.003)
Spells		−0.004	0.001	−0.005
		(0.010)	(0.007)	(0.008)
Spline 1		0.000	0.000	−0.000
		(0.000)	(0.000)	(0.000)
Spline 2		−0.000	−0.000	−0.000
		(0.000)	(0.000)	(0.000)
Spline 3		0.000	0.000*	0.000
		(0.000)	(0.000)	(0.000)
Constant		−48.021***	−35.764***	−38.627***
		(4.127)	(3.113)	(2.981)
ρ		.220	.203	.092
*N*		7,560	12,600	12,600
Log likelihood		−1,333.3	−1,996.0	−2,153.4

*Note.* Entries are coefficients and standard errors from random-effects binary time-series–cross-section (BTSCS) models, with presence/absence of an attack from a specific sender on a specific target as the dependent variable. The number of observations is the number of party dyads × days × hours (one hour drops because of the lagged variables). There are twelve dyads (four parties) in 2002 and twenty dyads (five parties) in 2006 and 2008.

† *p* < .1. *p < .05. **p < .01. ***p < .001.

There is strong support for H1 in the three models. All three coefficients are positive and highly significant. Transforming them into odds ratios suggests that an attack from A on B increases the odds of a subsequent attack from B on A by somewhere between 47 and 80 percent. This implies that direct retaliation is a substantively important driver of the campaign behavior of political parties also in a multiparty PR system.

By contrast, no significant effect is found for H2a. Attacks against a specific party are no more likely if the sender has come under attack from any other party. Whereas all the coefficients are positive, the statistical significance is too low to give us much confidence that the effects are not due to chance. Therefore, we cannot confirm the first expectation derived from our second hypothesis.

Next, we use the same data set to run three models that specify the presence of an attack by the first party in each directed party dyad on *any party but the second party* as the dependent variable (Table 3). This allows us to examine H2b, that is, whether attacks by a specific party will make it more likely that the targeted party attacks any other party except the attacker. As it is plausible that parties’ attack behavior is driven by attacks from other parties than the first party in the dyad, we control for the influence of these attacks.

**Table 3.** Explaining Attacks on Targets Outside Dyad.

	Hypothesis	2002	2006	2008
Attack from other party within dyad (t − 1)	H2b	0.325*	0.172	0.291**
		(0.133)	(0.114)	(0.109)
Attack from any party outside dyad (t − 1)	*control*	−0.067	−0.177	0.045
		(0.194)	(0.150)	(0.139)
TV debate		0.654***	0.313**	0.824***
		(0.138)	(0.099)	(0.093)
Left–right distance (sender–target)		−0.057	−0.029	−0.077
		(0.102)	(0.079)	(0.072)
Coalition parties		1.052*	1.342*	0.353
		(0.468)	(0.602)	(0.504)
Size ratio (sender–target)		−0.521***	−0.154**	−0.576***
		(0.140)	(0.053)	(0.112)
Hour		9.910***	8.240***	7.259***
		(0.621)	(0.449)	(0.417)
Hour (squared)		−0.655***	−0.554***	−0.489***
		(0.041)	(0.030)	(0.027)
Hour (cubed)		0.013***	0.012***	0.010***
		(0.001)	(0.001)	(0.001)
Day		0.005	0.007**	0.001
		(0.003)	(0.003)	(0.002)
Spells		−0.074	−0.412***	−0.259***
		(0.110)	(0.081)	(0.070)
Spline 1		−0.002	−0.012***	−0.006***
		(0.003)	(0.002)	(0.002)
Spline 2		0.001	0.003***	0.001***
		(0.001)	(0.001)	(0.000)
Spline 3		−0.000	−0.000***	−0.000
		(0.000)	(0.000)	(0.000)
Constant		−47.278***	−38.912***	−33.656***
		(3.041)	(2.205)	(2.066)
ρ		.075	.131	.117
*N*		7,560	12,600	12,600
Log likelihood		−2,137.2	−3,528.3	−3,723.6

*Note.* Entries are coefficients and standard errors from random-effects binary time-series–cross-section (BTSCS) models, with presence/absence of an attack from a specific party on any party as the dependent variable. The number of observations is the number of party dyads × days × hours (one hour drops because of the lagged variables). There are twelve dyads (four parties) in 2002 and twenty dyads (five parties) in 2006 and 2008.

* *p* < .05. **p < .01. ***p < .001.

The models give some support for the second implication of H2. At least in the 2002 and 2008 campaigns, attacks from A on B had an effect on the likelihood of B attacking parties other than A. Both coefficients are positive and significant. The odds ratios are 1.38 (2002) and 1.34 (2008), respectively, suggesting that an attack by a specific party increased the odds of the target attacking any other party by more than a third.

The coefficient for the 2006 campaign is considerably smaller (a value of 0.17 translates into an odds ratio of 1.18) and comes with a *p* value of 0.13. While we can thus be relatively confident that some of the patterns implied by H2 were present in 2002 and 2008, the evidence is less conclusive for 2006.

In five out of six models in Tables 2 and 3, TV debates have a strong impact on the probability of parties attacking each other. The coefficients of around 0.7 or 0.8 (in 2002 and 2008) translate into odds ratios of around 2 or larger, suggesting that the odds of an attack on a party at least double on days when TV debates featuring that party’s leader take place (the effects are smaller for the 2006 campaign).

The dyad-specific control variables in Tables 2 and 3 yield some further insights. First, the left–right distance between sender and target is not statistically significant in any of the models. This may have to do with the absence of preelectoral coalitions in Austria where parties typically fight elections on their own.

The predictor for the incumbent coalition, by contrast, is significant in several specifications. Remember that in 2002 and 2006, the sitting government was of the center–right (ÖVP-FPÖ and ÖVP-BZÖ, respectively), whereas a grand coalition of SPÖ and ÖVP was in place in 2008. The results from both sets of models suggest that the back and forth between these two parties was the dominant line of conflict during all campaigns, no matter the composition of the incumbent government. With regard to direct retaliation (Table 2), the coalition predictor is significant only for the 2008 grand coalition. The overall level of negativity was thus considerably higher in the SPÖ-ÖVP and ÖVP-SPÖ dyads. In Table 3, the predictors are positive and significant in 2002 and 2006 (when the SPÖ was in opposition), meaning that attacks from the coalition partner increased the probability of an *attack on another party*, thus suggesting that the main line of conflict coincided with the government—opposition divide. Again, the dyads comprised of the two traditional major parties are the most negative in those two campaigns.

Finally, the size ratio (the ratio of sender size to target size) is negative and significant in several model specifications, indicating that there is a tendency for larger parties to ignore their smaller competitors (and for smaller ones to focus on larger competitors). This effect is weaker (or nonexistent) for direct retaliation in 2002 and 2006 (Table 2)—the two campaigns when a small party participated in government (FPÖ and BZÖ, respectively).

Even a cursory look at the time effects in the six models suggests that attacks strongly follow a daily cycle (see the online appendix). The hour variables we use to model this dynamic are highly significant and thus capture the attack interactions that occur by mere temporal coincidence (even though some of them may have been genuine sequences of attacks and counterattacks). Furthermore, it is interesting to note that it is the inclusion of the hour variables that renders the spells and splines covariates insignificant in Table 2. Most of the time dependency picked up by these predictors thus relates to the daily increase and decrease in communication activity rather than to macrotrends over the course of the campaign.

**Figure 1.**
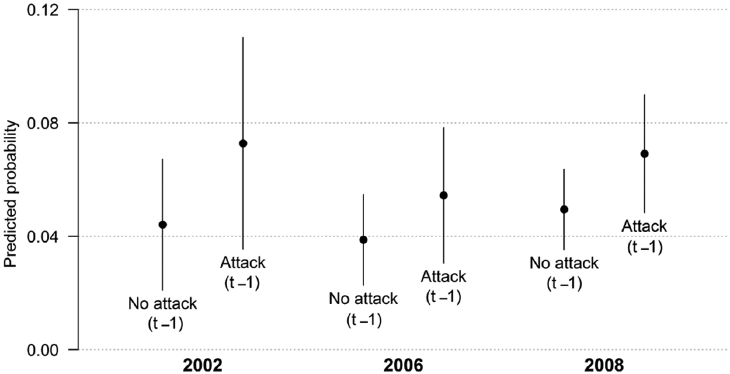
Predicted probabilities of attacks on target within dyad (direct retaliation, H1). *Note.* Calculation of predicted probabilities based on models reported in Table 2, assuming *u_i_* = 0. All other covariates kept at their means (continuous variables) or modes (categorical variables).

To present our main findings more intuitively, Figure 1 reports the predicted probabilities associated with H1. For the calculation of these quantities, the random part of the model capturing variation in the level of negative campaigning between party dyads (*u_i_*) was assumed to be zero.

In 2002, the increase in the probability of a targeted counterattack is from 4.4 to 7.8 percent, in 2006, it is from 3.9 to 5.4 percent, and in 2008, it is from 4.9 to 6.9 percent. While these may appear to be small numbers, bear in mind that most parties do not attack all other parties at every hour during the campaign. While the back and forth of attacks and counterattacks between specific parties at certain times can be quite intense, most parties choose to ignore some of their competitors much of the time and only attack them when they feel pressured to do so.

**Figure 2.**
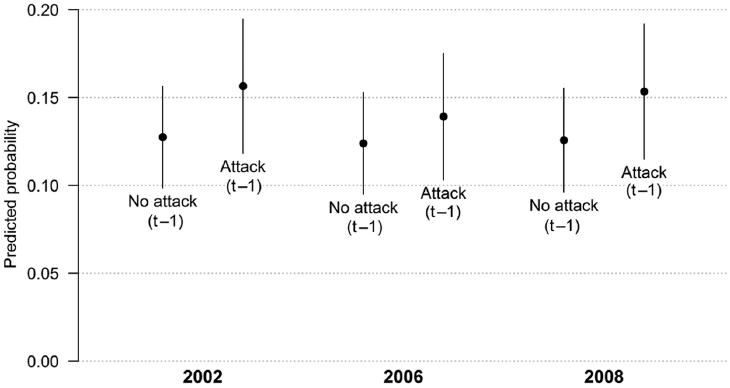
Predicted probabilities of attack on targets outside dyad (H2b). *Note.* Calculation of predicted probabilities based on models reported in Table 3, assuming *u_i_* = 0. All other covariates kept at their means (continuous variables) or modes (categorical variables).

Figure 2 plots the effects for the second part of H2. Here, we find that the baseline probabilities are somewhat higher. This is not surprising as the dependent variable now includes all attacks by a party, irrespective of who the target is. The effect sizes themselves, however, are rather modest, with increases in the predicted probabilities from around 12.5 to around 15 percent.

## Conclusion

The growing literature on negative campaigning outside the United States (Nai and Walter 2015) demonstrates that attacks are a central campaign strategy also in multiparty systems with PR, even though the incentives that guide party behavior in such systems are very different. In what constitutes (with the possible exception of de Nooy and Kleinnijenhuis 2013) the first dynamic study of the logic of retaliation in a multiparty system, we report not only evidence for the commonalities but also the differences in attack behavior between two-party, winner-takes-all competition and multiparty PR systems. In both systems, actors who have been attacked display a tendency to retaliate against the perpetrator of the attack. In addition, we find that in multiparty systems, negative messages against one party are, in some cases, also reactions to attacks from a different competitor.

Our analysis uses press releases as a data source, thus being able to observe campaign communication at very short time intervals. Yet it is important to consider that party communication via press releases must conform to the logic of journalistic news selection to reach the voters. This caveat notwithstanding, our study makes three contributions:

First, drawing on the literature on negativity bias in information processing, we provide a more solid theoretical argument than previous research for why attacks by one political actor often elicit counterattacks. In the light of this research, responding in kind is indeed a rational strategy: The zero-sum nature of elections means that relative losses in support can only be compensated by attacking a competitor.

Second, the zero-sum argument has different implications for two-party and multiparty competition. Whereas retaliation is the only plausible response strategy in two-party systems, multiparty systems allow for more complex patterns of interactions between parties.

Third, we draw the most high-definition picture of the use of negative messages during election campaigns to date. By observing attacks and counterattacks at hourly intervals, we not only leverage a rich data source. The close temporal proximity between individual messages (and different statistical controls) also gives us greater confidence that the responses we observe are, in fact, caused by the attacks immediately preceding them.

Substantively, our results show that retaliation is a strong empirical phenomenon in multiparty elections. Yet the analyses also indicate that party interactions do not exclusively follow the logic of retaliation. Parties also react to attacks by targeting other parties than those striking first. To be sure, the evidence for the tit-for-tat strategy is more robust than that for the more complex reaction patterns involving more than two parties. The latter empirical phenomenon must therefore be considered secondary in importance. We suggest that our findings may be driven by asymmetries in party size or strategic considerations related to postelectoral bargaining. Identifying the precise conditions under which parties attack each other in multiparty competition is thus one of the foremost tasks for future research on negative campaigning.

## Supplementary Material

Supplementary material

## Notes

1. Lau et al. (2007) report that thirty-three out of forty studies examining the impact of negative messages on the attacker find such a *backlash effect*.

2. Note that we do not aim to test the presence of negativity bias in political communication. Rather, negativity bias is part of the argument that portrays retaliation as a rational strategy.

3. In terms of substance, these two hypotheses mean similar things. Yet we separate them here because they demand two different empirical strategies.

4. The only exception we are aware of is Benoit’s *functional analysis* of campaign communication, which also considers negative references to other candidates or parties (e.g., Cho and Benoit 2006).

5. Excluding the hours between midnight and 8 a.m. eliminates only a tiny number of attacks from our sample (five in 2002, seven in 2006, and three in 2008).

6. We have also run our models with two-hour and three-hour lags. The substantive results remain unchanged. The significant effects, however, are mostly present for the covariates lagged by one hour.
