# Supplementary material for: Negative Campaigning and the Logic of Retaliation in Multiparty Competition
Source: Int J Press Polit. 2016 Jan 29;21(2):253–72. doi: 10.1177/1940161215626566 (PMC5111739; doi:10.1177/1940161215626566)
Supplement: Supplementary material [file IJPP-Online_Appendix.pdf]

Appendix: Negative campaigning and the logic of retaliation in multi-party competition

In this appendix we offer the following additional information:

1) Controls for time dependency in the distribution of attacks
2) Descriptive statistics of the independent variables
3) Fixed effects specifications of our regression models

1) Time dependency in the distribution of attacks

It should be noted that the timing of attacks is not distributed uniformly throughout the day but follows standard working hours, editorial deadlines, and certain campaign events. As Figure A1 shows, attacks pick up after 8 am, reaching their peak around 1 pm before declining to the single digits around 8 pm. The small upticks in the three distributions later in the evening are mostly caused by party operatives reacting to televised debates between party leaders which have since 1970 been a regular feature of Austrian election campaigns (and are broadcast at prime time). This is why we include a control variable that captures whether a party was taking part in a TV debate on a specific day, assuming that this will increase the level of negativity for that party (both, as a sender and receiver of attacks).

Figure A1: The daily cycle of attacks (total number of attacks per hour)

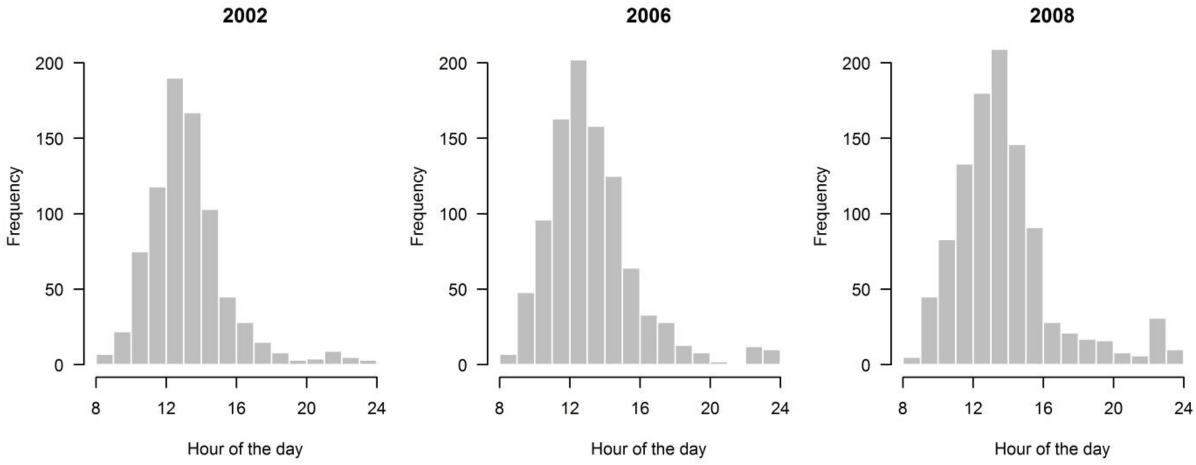

In order to account for the strong curvilinear effects over the course of a day, the models include as predictors the hour of the attack in its linear, squared, and cubed form. Since the identification of relationships between attacks and counter-attacks in this analysis rests on temporal sequence, this modelling strategy places strong demands on the core variables of interest. Yet, this is a necessary step to ensure that our results are not driven by mere temporal coincidence due to the daily ebb and flow of attack releases.

2) Descriptive statistics

The tables below present descriptive statistics for the variables used in the multivariate analyses in Tables 2 and 3 in the main text.

Table A1: Descriptive statistics for analyses of targeted attacks (H1 & H2a)

Year	Variable	N	Mean	SD	Min	Max
2002	Attack (dependent variable)	7560	0.076	0.265	0	1
	Attack from targeted party (t-1)	7560	0.077	0.266	0	1
	Attack from any non-targeted party (t-1)	7560	0.142	0.349	0	1
	TV debate	7560	0.071	0.258	0	1
	Left-right distance (sender – target)	7560	3.442	1.724	0.920	5.800
	Coalition parties	7560	0.167	0.373	0	1
	Size ratio (sender / target)	7560	1.450	1.143	0.321	3.119
	Weekend	7560	0.286	0.452	0	1
	Hour	7560	16.000	4.321	9	23
	Hour (squared)	7560	274.667	139.256	81	529
	Hour (cubed)	7560	4992.000	3553.541	729	12167
	Day	7560	21.500	12.122	1	42
2006	Attack (dependent variable)	12600	0.060	0.237	0	1
	Attack from targeted party (t-1)	12600	0.060	0.237	0	1
	Attack from any non-targeted party (t-1)	12600	0.138	0.345	0	1
	TV debate	12600	0.095	0.294	0	1
	Left-right distance (sender – target)	12600	4.000	2.169	0.840	7.500
	Coalition parties	12600	0.100	0.300	0	1
	Size ratio (sender / target)	12600	2.520	3.271	0.085	11.723
	Weekend	12600	0.286	0.452	0	1
	Hour	12600	16.000	4.321	9	23
	$Hour^2$	12600	274.667	139.252	81	529
	$Hour^3$	12600	4992.000	3553.447	729	12167
	Day	12600	21.500	12.121	1	42
2008	Attack (dependent variable)	12600	0.063	0.244	0	1
	Attack from targeted party (t-1)	12600	0.063	0.243	0	1
	Attack from any non-targeted party (t-1)	12600	0.152	0.359	0	1
	TV debate	12600	0.095	0.294	0	1
	Left-right distance (sender – target)	12600	3.643	2.143	0.643	6.643
	Coalition parties	12600	0.100	0.300	0	1
	Size ratio (sender / target)	12600	1.535	1.424	0.199	5.027
	Weekend	12600	0.286	0.452	0	1
	Hour	12600	16.000	4.321	9	23
	Hour (squared)	12600	274.667	139.252	81	529
	Hour (cubed)	12600	4992.000	3553.447	729	12167
	Day	12600	21.500	12.121	1	42

Table A2: Descriptive statistics for analyses of non-targeted attacks (H2b)

Year	Variable	N	Mean	SD	Min	Max
2002	Attack (dependent variable)	7560	0.141	0.348	0	1
	Attack from specific non-targeted party (t-1)	7560	0.077	0.266	0	1
	Attack from any other party (t-1)	7560	0.142	0.349	0	1
	TV debate	7560	0.071	0.258	0	1
	Left-right distance (sender–target)	7560	3.442	1.724	0.920	5.800
	Coalition parties	7560	0.167	0.373	0	1
	Size ratio (sender / target)	7560	1.450	1.143	0.321	3.119
	Hour	7560	16.000	4.321	9	23
	Hour (squared)	7560	274.667	139.256	81	529
	Hour (cubed)	7560	4992.000	3553.541	729	12167
	Day	7560	21.500	12.122	1	42
2006	Attack (dependent variable)	12600	0.139	0.351	0	7
	Attack from specific non-targeted party (t-1)	12600	0.060	0.237	0	1
	Attack from any other party (t-1)	12600	0.138	0.345	0	1
	TV debate	12600	0.095	0.294	0	1
	Left-right distance (sender–target)	12600	4.000	2.169	0.840	7.500
	Coalition parties	12600	0.100	0.300	0	1
	Size ratio (sender / target)	12600	2.520	3.271	0.085	11.723
	Hour	12600	16.000	4.321	9	23
	Hour (squared)	12600	274.667	139.252	81	529
	Hour (cubed)	12600	4992.000	3553.447	729	12167
	Day	12600	21.500	12.121	1	42
2008	Attack (dependent variable)	12600	0.155	0.393	0	9
	Attack from specific non-targeted party (t-1)	12600	0.063	0.243	0	1
	Attack from any other party (t-1)	12600	0.152	0.359	0	1
	TV debate	12600	0.095	0.294	0	1
	Left-right distance (sender–target)	12600	3.643	2.143	0.643	6.643
	Coalition parties	12600	0.100	0.300	0	1
	Size ratio (sender / target)	12600	1.535	1.424	0.199	5.027
	Hour	12600	16.000	4.321	9	23
	Hour (squared)	12600	274.667	139.252	81	529
	Hour (cubed)	12600	4992.000	3553.447	729	12167
	Day	12600	21.500	12.121	1	42

3) Fixed-effects models

Below we report fixed-effects versions of the random-effects models in the main text. As can be seen, the results are extremely similar, with no substantive differences in the signs or significance of the core coefficients. Note, however, that predictors that are constant within party dyads (left–right distance, coalition parties, size ratio) are dropped from the analysis.

Table A3: Explaining targeted reactions to attacks

	Hypothesis	2002	2006	2008
Attack from targeted party (t-1)	H1	0.567***	0.364**	0.413***
		(0.134)	(0.126)	(0.123)
Attack from any non-targeted party (t-1)	H2 (part one)	0.050	0.169	0.179
		(0.137)	(0.118)	(0.112)
TV debate		0.702***	0.143	0.723***
		(0.172)	(0.139)	(0.119)
Hour		9.614***	7.415***	7.795***
		(0.832)	(0.639)	(0.607)
Hour (squared)		-0.636***	-0.499***	-0.518***
		(0.055)	(0.043)	(0.040)
Hour (cubed)		0.013***	0.010***	0.011***
		(0.001)	(0.001)	(0.001)
Day		0.004	0.008*	0.001
		(0.004)	(0.004)	(0.003)
Spells		-0.004	0.001	-0.005
		(0.010)	(0.007)	(0.008)
Spline 1		0.000	0.000	-0.000
		(0.000)	(0.000)	(0.000)
Spline 2		-0.000	-0.000	0.000
		(0.000)	(0.000)	(0.000)
Spline 3		0.000	0.000*	0.000
		(0.000)	(0.000)	(0.000)
Party-dyad fixed effects		Yes	Yes	Yes
N		7560	11970	12600
McFadden's R2		0.232	0.176	0.157
Log likelihood		-1307.0	-1965.1	-2106.8

Note: Entries are coefficients and standard errors from fixed effects BTSCS models with presence/absence of an attack from a specific party on another specific party as the dependent variable. The number of observations is the number of party dyads × days × hours (1 hour drops because of the lagged variables). There are 12 dyads (four parties) in 2002 and 20 dyads in 2006 and 2008 (one dyad drops in 2006 due to lack of variation in the dependent variable). * $p < 0.05$, ** $p < 0.01$, *** $p < 0.001$.

Table A4: Explaining non-targeted reactions to attacks

	Hypothesis	2002	2006	2008
Attack from specific non-targeted party (t-1)	H2 (part two)	0.364**	0.177	0.291**
		(0.134)	(0.114)	(0.109)
Attack from any other party (t-1)	control	-0.063	-0.178	0.042
		(0.194)	(0.150)	(0.139)
TV debate		0.659***	0.313**	0.826***
		(0.138)	(0.099)	(0.093)
Hour		9.899***	8.279***	7.309***
		(0.621)	(0.449)	(0.417)
Hour (squared)		-0.654***	-0.556***	-0.492***
		(0.041)	(0.030)	(0.028)
Hour (cubed)		0.013***	0.012***	0.010***
		(0.001)	(0.001)	(0.001)
Day		0.005	0.007**	0.001
		(0.003)	(0.003)	(0.002)
Spells		-0.067	-0.406***	-0.254***
		(0.110)	(0.081)	(0.070)
Spline 1		-0.002	-0.012***	-0.006***
		(0.003)	(0.002)	(0.002)
Spline 2		0.000	0.003***	0.001***
		(0.001)	(0.001)	(0.000)
Spline 3		-0.000	-0.000***	-0.000
		(0.000)	(0.000)	(0.000)
Party-dyad fixed effects		Yes	Yes	Yes
N		7560	12600	12600
McFadden's R2		0.257	0.232	0.209
Log likelihood		-2074.8	-3425.0	-3617.1

Note: Entries are coefficients and standard errors from fixed effects BTSCS models with presence/absence of an attack from a specific party on any party as the dependent variable. The number of observations is the number of party dyads × days × hours (1 hour drops because of the lagged variables). There are 12 dyads (four parties) in 2002 and 20 dyads in 2006 and 2008 (one dyad drops in 2006 due to lack of variation in the dependent variable).
* $p < 0.05$, ** $p < 0.01$, *** $p < 0.001$.
